# GLP-1 receptor agonists in diabetes and weight loss: the double-edged sword of innovation and risks

**DOI:** 10.3389/fcdhc.2024.1530811

**Published:** 2025-01-09

**Authors:** Ioannis Ilias, Lina Zabuliene, Manfredi Rizzo

**Affiliations:** ^1^ Department of Endocrinology, Hippokration General Hospital, Athens, Greece; ^2^ Institute of Clinical Medicine, Faculty of Medicine, Vilnius University, Vilnius, Lithuania; ^3^ School of Medicine, Department of Health Promotion, Mother and Child Care, Internal Medicine and Medical Specialties (Promise), University of Palermo, Palermo, Italy

**Keywords:** glucagon-like peptide-1 receptor agonists, obesity, weight loss, diabetes, adverse drug reaction, pancreatitis, nonarteritic anterior ischemic optic neuropathy

In recent years, glucagon-like peptide-1 (GLP-1) receptor agonists have gained widespread popularity not only as effective agents in the management of type 2 diabetes but also for their beneficial impact on weight loss. Additionally, this surge in interest has been underpinned by their pleiotropic effects and has spurred further investigation into their potential applications beyond diabetes care. However, the expanding adoption of GLP-1 receptor agonists also brings with it a set of challenges and complications. With promising therapeutic effects comes an increased scrutiny on side effects.

GLP-1 receptor agonists mimic the action of the native GLP-1 peptide, enhancing insulin secretion, suppressing glucagon release, slowing gastric emptying, and increasing satiety. Initially introduced to treat diabetes, they were also found to have an important effect on weight loss; a paradigm shift in the medical management of obesity may - in fact - be imminent, per the opinion of medical and non-medical pundits (1–3). Drugs like semaglutide, liraglutide or tirzepatide (a dual GLP-1 and GIP analog) have led to significant weight reduction of participants in clinical trials and in real-world settings, making them popular for patients and physicians alike.

The success of GLP-1 receptor agonists in weight management lies in their multifaceted mechanisms, which address complex physiological pathways of hunger, satiety, and glucose metabolism. These drugs target not only the pancreas and gut but also the brain’s hypothalamic appetite-regulating centers. Some of the effects of GLP-1 analogs may be attributed to their actions on central nervous system neurotransmitter secretion/action; they enhance gamma-aminobutyric acid (GABA) activity, restore dopaminergic activity and act in parallel with peptide YY (PYY) (4–6). In this sense, GLP-1 analogs are redefining the therapeutic approach to metabolic disease by not only addressing glycemic control but also influencing body weight and cardiovascular health. Currently, long-term consequences of GLP-1 analog use for over 1.5 -2 years look promising; in published meta-analyses, reduced cardiovascular morbidity and mortality have been noted, whereas no effect on the appearance of gastrointestinal neoplasia has been observed (7, 8).

The pleiotropic effects of GLP-1 analogs foster ongoing research into new indications. Evidence suggests these drugs may also have potential cardiovascular benefits, with studies indicating reduced cardiovascular risk among people with type 2 diabetes who use GLP-1 receptor agonists (9–12). Additionally, studies have explored the impact of GLP-1 receptor agonists on liver health, including potential benefit in the treatment of metabolic dysfunction associated fatty liver disease (MASLD) (13, 14). The anti-inflammatory and neuroprotective properties of GLP-1 analogs are also being investigated, with implications for neurodegenerative conditions like Alzheimer’s disease (15–17). Such research raises the possibility of broader therapeutic applications for GLP-1 receptor agonists, as their influence extends beyond traditional diabetes and weight management.

As exciting as these advancements are, they raise critical questions about society’s preference for pharmacological solutions over traditional lifestyle interventions like diet and exercise. In the mindset of many patients and practitioners, the allure of weight-loss medications may indeed outweigh the need for lifestyle changes, despite evidence that diet and physical activity are fundamental components of sustainable health. Surveys of internet use worldwide have indicated a frank increase in searches for these new drugs (18, 19). However, it has also been revealed that people are more interested in pharmaceutical solutions for weight loss than in making significant lifestyle modifications. This trend is fueled by the relative ease and accessibility of medications, coupled with the perception that they can yield rapid, visible results (traditional and social media and statements by “celebrities” may add to this trend).

Pharmaceutical companies have responded to this demand with a pipeline of weight-loss drugs, many of which are based on the mechanisms of GLP-1 receptor agonists. This focus on pharmacotherapy, however, risks overshadowing the essential role of non-pharmacological interventions in chronic disease prevention and management. It is important for healthcare providers to emphasize that while medications like GLP-1 analogs can play a valuable role in treatment plans, they are not substitutes for a balanced diet, regular physical activity, and other lifestyle modifications.

While GLP-1 analogs are largely considered safe, their increasing usage has drawn attention to rare but significant adverse events. Nonarteritic anterior ischemic optic neuropathy (NAION), an idiopathic ischemic injury to the optic nerve head, marked by sudden, painless vision loss in the eye and swelling of the optic disc, has been reported in association with GLP-1 receptor agonists’ use, though such cases remain rare (20). The mechanisms by which GLP-1 analogs might trigger NAION are not yet fully understood, and further investigation is necessary to determine whether a causal relationship exists. In clinical practice, patients experiencing visual disturbances while on GLP-1 analog therapy should be evaluated promptly, with consideration given to discontinuing the medication if a link is suspected.

Pancreatitis is a known potential risk associated with GLP-1 receptor agonists. Although the absolute risk is low, pancreatitis can be severe and even life-threatening. GLP-1 receptors are expressed in the pancreas, which has led researchers to hypothesize that these drugs may influence pancreatic function in ways that could predispose certain individuals to inflammation. Studies on this association have yielded mixed to negative results (21–23), and while there is no definitive evidence linking GLP-1 receptor agonists to a higher incidence of pancreatitis, it remains a listed adverse effect.

Other significant adverse events, possibly linked to the use of GLP-1 analogs, include muscle mass loss, dermatological/hypersensitivity reactions, increased heart rate, reduced left ventricular ejection fraction and depression (24–26).

The emergence of these safety concerns reinforces the importance of individualized patient assessment before prescribing GLP-1 receptor agonists, particularly for those with a history of pancreatic disorders or other risk factors. Healthcare providers must engage in thorough discussions with patients, weighing the potential benefits against the risks and ensuring that patients understand the signs and symptoms of adverse events. The advent of GLP-1 receptor agonists represents a milestone in the treatment of diabetes and obesity, but it also presents a challenge in balancing the drive for innovation with the need for patient safety. These medications, like many in the pharmaceutical landscape, are not devoid of risks, and their popularity should not detract from the importance of comprehensive, individualized care. While GLP-1 analogs offer much promise, they also underscore the reality that pharmacotherapy alone cannot address the root causes of chronic metabolic diseases. As we move forward, it will be crucial to develop a nuanced approach to the prescription of GLP-1 receptor agonists and other weight-loss drugs. Educating patients about the importance of lifestyle modification, as well as the potential risks of pharmacotherapy, will remain essential. Additionally, further research is needed to clarify the mechanisms underlying adverse events and to identify which patients may be at higher risk (27).

GLP-1 receptor agonists have transformed the landscape of diabetes and obesity management, offering significant benefits that extend beyond glycemic control. However, the increasing reliance on pharmacological solutions over lifestyle interventions reflects a broader societal trend that risks de-emphasizing the role of diet and exercise in managing chronic diseases. The potential for adverse effects like NAION and pancreatitis, although rare, serves as a reminder of the complexities of drug therapy and the need for careful patient selection and monitoring. In our pursuit of medical advancements, the allure of innovative pharmacotherapies like GLP-1 analogs should be balanced with a commitment to safe and responsible practice. As healthcare providers, our responsibility is not only to embrace the tools that modern medicine provides but also to remain vigilant in safeguarding patient health through informed, evidence-based care. The promise of GLP-1 analogs is undeniable, yet so is the need for caution and judicious use in our quest to improve patient outcomes.

## References

[1] Ahrén B. Paradigm shift in the management of metabolic diseases-next-generation incretin therapy. Endocrinology. (2023) 164:bqad166. doi: 10.1210/endocr/bqad166 37951841

[2] Gong B Yao Z Zhou C Wang W Sun L Han J. Glucagon-like peptide-1 analogs: Miracle drugs are blooming? Eur. J. Med. Chem. (2024) 269:116342. doi: 10.1016/j.ejmech.2024.116342 38531211

[3] Anonymous. One jab to treat them all Vol. 453. London, UK: The Economist (2024) p. 14–6.

[4] Manavi MA. Neuroprotective effects of glucagon-like peptide-1 (GLP-1) analogues in epilepsy and associated comorbidities. Neuropeptides. (2022) 94:102250. doi: 10.1016/j.npep.2022.102250 35561568

[5] Ferrari F Moretti A Villa RF. Incretin-based drugs as potential therapy for neurodegenerative diseases: current status and perspectives. Pharmacol. Ther. (2022) 239:108277. doi: 10.1016/j.pharmthera.2022.108277 36064147

[6] Verbiest A Wauters L Vanuytsel T. Enterohormone therapy for short bowel syndrome. Curr. Opin. Endocrinol. Diabetes Obes. (2022) 29:207–18. doi: 10.1097/MED.0000000000000710 35034035

[7] Alexander JT Staab EM Wan W Franco M Knitter A Skandari MR. The longer-term benefits and harms of glucagon-like peptide-1 receptor agonists: a systematic review and meta-analysis. J. Gen. Intern. Med. (2022) 37:415–38. doi: 10.1007/s11606-021-07105-9 PMC8810987 34508290

[8] Figlioli G Piovani D Peppas S Pugliese N Hassan C Repici A. Glucagon-like peptide-1 receptor agonists and risk of gastrointestinal cancers: A systematic review and meta-analysis of randomized controlled trials. Pharmacol. Res. (2024) 208:107401. doi: 10.1016/j.phrs.2024.107401 39251099

[9] Tirandi A Montecucco F Carbone F Liberale L. Role of glucagon-like peptide-1 receptor agonists in the treatment of obesity, cardiovascular disease, and cerebrovascular disease. Pol. Arch. Intern. Med. (2024) 134:16658. doi: 10.20452/pamw.16658 38226456

[10] Muzurović E Yumuk VD Rizzo M. GLP-1 and dual GIP/GLP-1 receptor agonists in overweight/obese patients for atherosclerotic cardiovascular disease prevention: Where are we now? J. Diabetes Complications. (2023) 37:108647. doi: 10.1016/j.jdiacomp.2023.108647 37952274

[11] Bechlioulis A Markozannes G Chionidi I Liberopoulos E Naka KK Ntzani EE. The effect of SGLT2 inhibitors, GLP1 agonists, and their sequential combination on cardiometabolic parameters: A randomized, prospective, intervention study. J. Diabetes Complications. (2023) 37:108436. doi: 10.1016/j.jdiacomp.2023.108436 36842186

[12] Janez A Muzurovic E Bogdanski P Czupryniak L Fabryova L Fras Z. Modern management of cardiometabolic continuum: from overweight/obesity to prediabetes/type 2 diabetes mellitus. Recommendations from the eastern and southern Europe diabetes and obesity expert group. Diabetes Ther. (2024) 5:1865–92. doi: 10.1007/s13300-024-01615-5 PMC11330437 38990471

[13] Rochoń J Kalinowski P Szymanek-Majchrzak K Grąt M. Role of gut-liver axis and glucagon-like peptide-1 receptor agonists in the treatment of metabolic dysfunction-associated fatty liver disease. World J. Gastroenterol. (2024) 30:2964–80. doi: 10.3748/wjg.v30.i23.2964 PMC11212696 38946874

[14] Abdelmalek MF Harrison SA Sanyal AJ. The role of glucagon-like peptide-1 receptor agonists in metabolic dysfunction-associated steatohepatitis. Diabetes Obes. Metab. (2024) 26:2001–16. doi: 10.1111/dom.15524 38511418

[15] Chen SD Chuang YC Lin TK Yang JL. Alternative role of glucagon-like Peptide-1 receptor agonists in neurodegenerative diseases. Eur. J. Pharmacol. (2023) 938:175439. doi: 10.1016/j.ejphar.2022.175439 36470445

[16] Hong CT Chen JH Hu CJ. Role of glucagon-like peptide-1 receptor agonists in Alzheimer’s disease and Parkinson’s disease. J. BioMed. Sci. (2024) 31:102. doi: 10.1186/s12929-024-01090-x 39501255 PMC11539687

[17] Crook H Edison P. Incretin mimetics as potential disease modifying treatment for Alzheimer’s disease. J. Alzheimers Dis. (2024) 101:S357–70. doi: 10.3233/JAD-240730 39422964

[18] Tselebis A Ilias I. Further research on internet searches for on- and off-label use of weight-loss medications. Aesthet Surg. J. (2023) 43:NP977–8. doi: 10.1093/asj/sjad258 37562032

[19] Raubenheimer JE Myburgh PH Bhagavathula AS. Sweetening the deal: an infodemiological study of worldwide interest in semaglutide using Google Trends extended for health application programming interface. BMC Global Public Health. (2024) 2:63. doi: 10.1186/s44263-024-00095-w 39681910 PMC11622965

[20] Hathaway JT Shah MP Hathaway DB Zekavat SM Krasniqi D Gittinger JW Jr. Risk of nonarteritic anterior ischemic optic neuropathy in patients prescribed semaglutide. JAMA Ophthalmol. (2024) 142:732–9. doi: 10.1001/jamaophthalmol.2024.2296 PMC11223051 38958939

[21] Sodhi M Rezaeianzadeh R Kezouh A Etminan M. Risk of gastrointestinal adverse events associated with glucagon-like peptide-1 receptor agonists for weight loss. JAMA. (2023) 330:1795–7. doi: 10.1001/jama.2023.19574 PMC10557026 37796527

[22] Pratley R Saeed ZI Casu A. Incretin mimetics and acute pancreatitis: enemy or innocent bystander? Curr. Opin. Gastroenterol. (2024) 40:404–12. doi: 10.1097/MOG.0000000000001057 38967917

[23] Masson W Lobo M Barbagelata L Lavalle-Cobo A Nogueira JP. Acute pancreatitis due to different semaglutide regimens: An updated meta-analysis. Endocrinol. Diabetes Nutr. (Engl Ed). (2024) 71:124–32. doi: 10.1016/j.endien.2024.03.012 38555109

[24] Mechanick JI Butsch WS Christensen SM Hamdy O Li Z Prado CM. Strategies for minimizing muscle loss during use of incretin-mimetic drugs for treatment of obesity. Obes. Rev. (2025) 26:e13841. doi: 10.1111/obr.13841 39295512 PMC11611443

[25] Salazar CE Patil MK Aihie O Cruz N Nambudiri VE. Rare cutaneous adverse reactions associated with GLP-1 agonists: a review of the published literature. Arch. Dermatol. Res. (2024) 316:248. doi: 10.1007/s00403-024-02969-3 38795152

[26] Tobaiqy M. A review of serious adverse events linked with GLP-1 agonists in type 2 diabetes mellitus and obesity treatment. Pharmacol. Rep. (2024) 76:981–90. doi: 10.1007/s43440-024-00629-x 39093550

[27] Gómez-Peralta F Abreu C Rizzo M. GLP-1 receptor agonists and SGLT2 inhibitors: The need to shed light on their safety risks real dimension and possible mechanisms. J. Diabetes Complications. (2023) 37:108553. doi: 10.1016/j.jdiacomp.2023.108553 37385011

